# Fully Attenuated *meq* and *pp38* Double Gene Deletion Mutant Virus Confers Superior Immunological Protection against Highly Virulent Marek’s Disease Virus Infection

**DOI:** 10.1128/spectrum.02871-22

**Published:** 2022-11-09

**Authors:** Aijun Sun, Xuyang Zhao, Xiaojing Zhu, Zhengjie Kong, Yifei Liao, Man Teng, Yongxiu Yao, Jun Luo, Venugopal Nair, Guoqing Zhuang, Gaiping Zhang

**Affiliations:** a College of Veterinary Medicine, Henan Agricultural University, Zhengzhou, Henan, People’s Republic of China; b International Joint Research Center of National Animal Immunology, College of Veterinary Medicine, Henan Agricultural University, Zhengzhou, Henan, People’s Republic of China; c Division of Infectious Disease, Department of Medicine, Brigham and Women's Hospital, Harvard Medical School, Boston, Massachusetts, USA; d Key Laboratory of Animal Immunology, Ministry of Agriculture and Rural Affairs & Henan Provincial Key Laboratory of Animal Immunology, Henan Academy of Agricultural Sciences, Zhengzhou, Henan, People’s Republic of China; e UK-China Centre of Excellence for Research on Avian Diseases, Henan Academy of Agricultural Sciences, Zhengzhou, Henan, People’s Republic of China; f Viral Oncogenesis Group,The Pirbright Institute, Pirbright, Surrey, United Kingdom; g Jiangsu Co-Innovation Center for the Prevention and Control of Important Animal Infectious Disease and Zoonoses, Yangzhou University, Yangzhou, People’s Republic of China; h UK-China Centre of Excellence for Research on Avian Diseases, The Pirbright Institute, Pirbright, Surrey, United Kingdom; University of Florida

**Keywords:** Marek’s disease virus, *meq*, lymphoid organ atrophy, *pp38*, gene deletion, vaccine

## Abstract

Marek’s disease virus (MDV) induces immunosuppression and neoplastic disease in chickens. The virus is controllable via an attenuated *meq* deletion mutant virus, which has the disadvantage of retaining the ability to induce lymphoid organ atrophy. To overcome this deficiency and produce more vaccine candidates, a recombinant MDV was generated from the highly virulent Md5BAC strain, in which both *meq* and a cytolytic replication-related gene, *pp38*, were deleted. Replication of the double deletion virus, Md5BAC Δ*meq*Δ*pp38*, was comparable with that of the parental virus *in vitro*. The double deletion virus was shown to be fully attenuated and to reduce lymphoid organ atrophy *in vivo*. Crucially, Md5BAC Δ*meq*Δ*pp38* confers superior protection against highly virulent virus compared with a commercial vaccine strain, CVI988/Rispens. Transcriptomic profiling indicated that Md5BAC Δ*meq*Δ*pp38* induced a different host immune response from CVI988/Rispens. In summary, a novel, effective, and safe vaccine candidate for prevention and control of MD caused by highly virulent MDV is reported.

**IMPORTANCE** MDV is a highly contagious immunosuppressive and neoplastic pathogen. The virus can be controlled through vaccination via an attenuated *meq* deletion mutant virus that retains the ability to induce lymphoid organ atrophy. In this study, we overcame the deficiency by generating *meq* and *pp38* double deletion mutant virus. Indeed, the successfully generated *meq* and *pp38* double deletion mutant virus had significantly reduced replication capacity *in vivo* but not *in vitro*. It was fully attenuated and conferred superior protection efficacy against very virulent MDV challenge. In addition, the possible immunological protective mechanism of the double deletion mutant virus was shown to be different from that of the gold standard MDV vaccine, CVI988/Rispens. Overall, we successfully generated an attenuated *meq* deletion mutant virus and widened the range of potential vaccine candidates. Importantly, this study provides for the first time the theoretical basis of vaccination induced by fully attenuated gene-deletion mutant virus.

## INTRODUCTION

Marek’s disease (MD) is an immunosuppressive and neurological disease of chickens induced by highly contagious Marek’s disease virus (MDV). MDV infection causes rapid development of T cell lymphomas, according to viral virulence and host susceptibility (1, 2). MD can be prevented and controlled by attenuated MDV-1 (*Gallid alphaherpesvirus* 2, GaHV-2), nonpathogenic MDV-2 (*Gallid alphaherpesvirus* 3, GaHV-3), and turkey herpesvirus (*Meleagrid alphaherpesvirus* 1, MeHV-1) vaccines (3, 4). However, the phenomenon of “leaky vaccination” may have allowed the evolution of MDV into strains of increasing virulence (5 to 7). Pathogenic MDVs have been classified into four pathotypes: mildly virulent (m), virulent (v), very virulent (vv), and very virulent plus (vv+). CVI988/Rispen is a fully attenuated vaccine, considered to be the most efficacious and the gold standard MDV vaccine. However, continuing outbreaks of MD indicate the need to develop novel and robust next-generation vaccines (3, 4).

Many specific deletion and insertion modifications to MDV genes have been explored (4, 8 to 10). For example, the MDV-specific *meq* gene encodes a 339 amino acid protein containing a basic leucine zipper (bZIP) domain that forms homodimers and also heterodimers by interacting with host-derived c-Jun or c-Fos proteins. The resulting complexes induce tumor formation through binding to viral and cellular genomes (11 to 14). Deletion of the *meq* gene in a range of virulent MDV strains completely disabled tumor induction, suggesting a critical role in virus-induced tumorigenesis (15). Furthermore, the *meq* deletion virus gives superior protection against challenge with highly virulent MDV strains, making it an excellent vaccine candidate (16 to 18). However, the *meq* deletion virus retains the ability to induce lymphoid organ atrophy, reducing its potential as a vaccine (19). Cell-culture passage-attenuated strains have been generated to overcome this disadvantage, but show reduced protective efficacy (20). Considering that the virus’s cytolytic replication capacity might be responsible for the induction of lymphoid organ atrophy, MDV double deletion mutants have been generated where *meq* plus a viral early cytolytic replication gene have been knocked out (9, 21).

MDV encodes over 100 genes, most of which are located on the unique long (U_L_) and unique short (U_S_) regions of the viral genome. The majority of the loci are homologous of herpes simplex virus (HSV-1) genes and are involved in viral genomic DNA replication, viral particle assembly, and morphogenesis (11). Over the last 2 decades, advances in genome editing techniques have accelerated MDV gene functional analysis. MDV gene deletion mutants have been generated using bacterial artificial chromosomes (BACs) and functional analysis of replication, and pathogenesis performed. Highly conserved genes in the U_L_ region, including glycoprotein B (*gB*), *gE*, *gI*, and *gM*, have been demonstrated to be essential for MDV replication *in vitro* (22 to 24). In contrast, gC was detrimental to MDV replication in cultured chicken embryonic fibroblast (CEF) cells (25). *U_S_3* and *U_L_13* sequences encode MDV serine/threonine protein kinases (26), which are highly conserved among alpha-herpesviruses but exert distinctive roles in MDV replication and pathogenesis (27 to 30). U_L_28 and U_L_33 have been found to be essential for MDV packaging and maturation (31). In addition, unique genes encoded by the IR/TR repeat regions have roles in viral cytolytic replication. For example, vIL8 (virus-encoded interleukin 8) is an MDV-encoded homolog of cellular IL-8, a C-X-C motif chemokine (32). Previous studies have shown that vIL8 is involved in pathogenesis and recruits B and CD4^+^CD25^+^ T cells to the infection site (33). The *vIL8* deletion mutant virus had reduced early cytolytic replication and did not induce lymphoid organ atrophy but still caused tumor formation (33, 34). A double deletion mutant virus, in which *meq* and *vIL8* were knocked out, showed reduced lymphoid organ atrophy and provided protection effects comparable to CVI988/Rispens (9). However, there remains an urgent need to develop more qualified vaccine candidates to combat the rapid evolution of MDV.

The MDV locus, *pp38*, is situated at the junction of the U_L_ and IR_L_ regions and encodes a polypeptide that is phosphorylated during pathogenesis (11). pp38 undergoes cytosolic phosphorylation by U_S_3 with an unclear molecular mechanism (29). Deletion of *pp38* in vv MDV did not affect *in vitro* replication but reduced early cytolytic replication and eliminated viral pathogenesis. However, the *pp38* deletion mutant still induced tumor formation, rendering it unsuitable as a vaccine candidate (35). The current study describes the generation of the double gene deletion mutant, Md5BAC Δ*meq*Δ*pp38*, using the BAC clone of Md5, a vv MDV strain. Pathogenic properties and immunological protection were evaluated. Md5BAC Δ*meq*Δ*pp38* had a replication capacity comparable with that of the parental virus *in vitro*. However, Md5BAC Δ*meq*Δ*pp38* was fully attenuated and conferred protection in a manner superior to that conferred by CVI988/Rispens. In addition, evaluation of immunological responses to Md5BAC Δ*meq*Δ*pp38* indicates a different immunological mechanism for MD prevention in chickens.

## RESULTS

### Successful generation of the Md5BAC Δ*meqΔpp38* double gene deletion and revertant mutant viruses.

**FIG 1:**
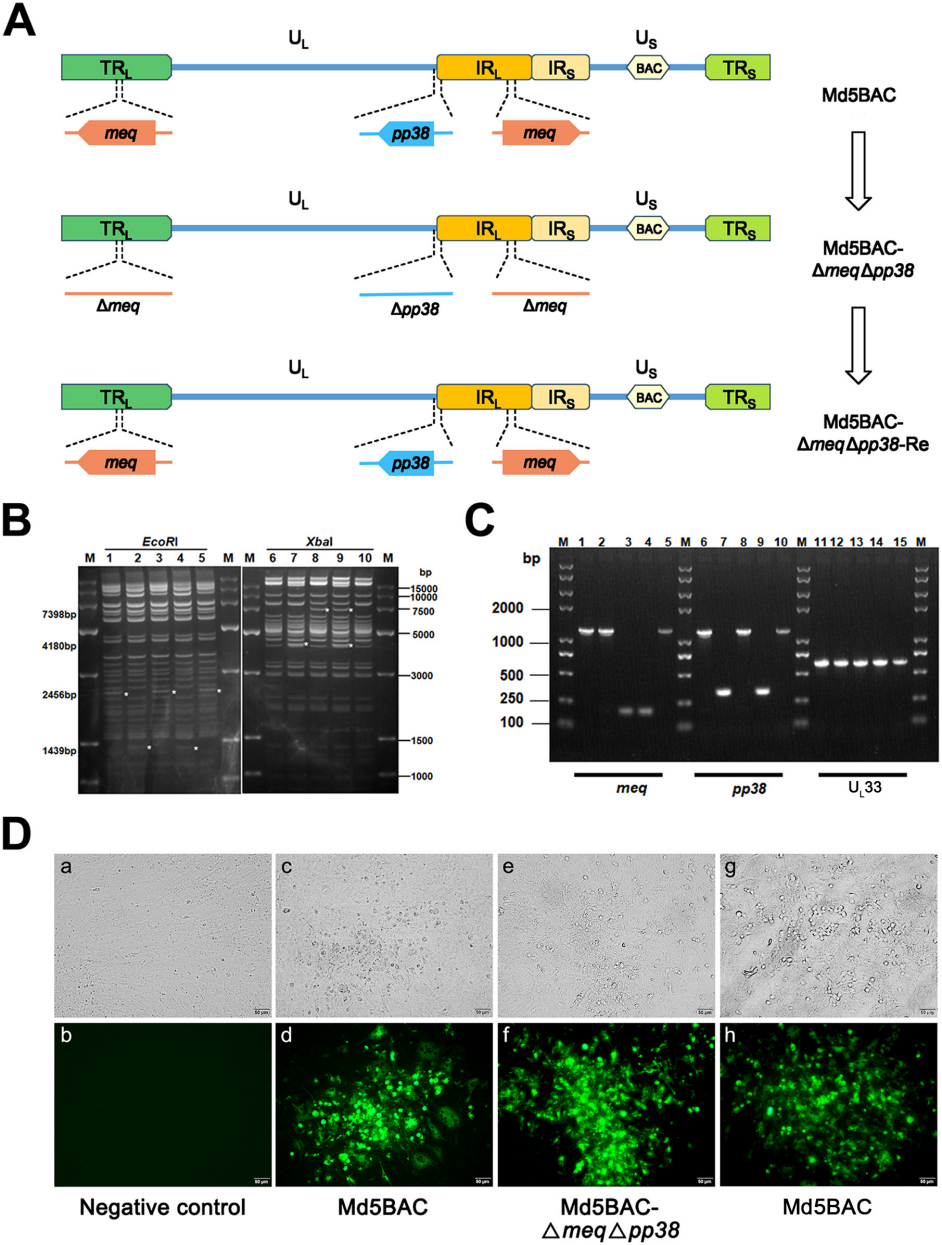
Construction and identification of the Md5BAC Δ*meq*Δ*pp38* double gene deletion and revertant mutant viruses. (A) Schematic representation of the MDV genome consisting of a unique long unique (U_L_) region and a short unique (U_S_) region, flanked by inverted internal and terminal repeat long (TR_L_, IR_L_) and short (TR_S_, IR_S_) regions. The *meq* gene is located in the IR_L_ and TR_L_ regions; the *pp38* gene is located at the junction of the U_L_ and IR_L_ regions. Entire ORFs of *meq* and *pp38* deletions are briefly outlined. (B) RFLP analysis of genomic DNAs from Md5BAC, Md5BAC Δ*meq*, Md5BAC Δ*pp38*, Md5BAC Δ*meq*Δ*pp38* and Md5BAC Δ*meq*Δ*pp38*-Re. DNA was digested with *Eco*RI and *Xba*l, and digested products were separated by 1% agarose gel electrophoresis and stained with Super GelRed (US Everbright, CA, USA). Lanes 1 and 6: Md5BAC; lanes 2 and 7: Md5BAC Δ*pp38*; lanes 3 and 8: Md5BAC Δ*meq*; lanes 4 and 9: Md5BAC Δ*meq*Δ*pp38*; lanes 5 and 10: Md5BAC Δ*meq*Δ*pp38*-Re. *, fragment size difference. (C) PCR analysis of *meq* and *pp38* deletion and revertant constructs. DNA of Md5BAC (lanes 1, 6, and 11), Md5BAC Δ*pp38* (lanes 2, 7, and 12), Md5BAC Δ*meq* (lanes 3, 8, and 13), Md5BAC Δ*meq*Δ*pp38* (lanes 4, 9. and 14), and Md5BAC Δ*meq*Δ*pp38*-Re (lanes 5, 10, and 15) were amplified by PCR using primers flanking *pp38*, *meq*, and *U_L_33*. DNA ladder and PCR product sizes are indicated in base pairs. M, 8 kb DNA ladder. (D) IFA analysis of MDV plaques in CEF. Images in e, f, g, and h represent the same cells as images a, b, c, and d, examined by fluorescence microscopy.

A previously reported two-step Red-mediated recombination method was used to delete the entire open reading frame (ORF) of the *pp38* gene from the Md5BAC Δ*meq* genome to produce the Md5BAC Δ*meq*Δ*pp38* mutant virus (Fig. 1A), and Md5BAC Δ*meq*Δ*pp38*-Re was generated by re-introduction of the *meq* and *pp38* genes. BAC DNAs were examined by RFLP analysis using *Xba*I and *Eco*RI enzymes to exclude the possibility of unexpected recombination events associated with the deletion process. Digestion of Md5BAC Δ*meq*Δ*pp38* with *Eco*RI resulted in 1,439 bp fragments with the loss of 2,456 bp (Fig. 1B, lane 4), and digestion with *Xba*I resulted in 7,398 bp and 4,180 bp fragments (Fig. 1B, lane 9). Digestion of Md5BAC (Fig. 1B, lanes 1 and 6), Md5BAC Δ*pp38* (Fig. 1B, lanes 2 and 7), Md5BAC Δ*meq* (Fig. 1B, lanes 3 and 8), and Md5BAC Δ*meq*Δ*pp38*-Re (lanes 5 and 10) with *Eco*RI and *Xba*I, respectively, is consistent with *in silico* predictions and indicating the absence of unexpected rearrangements in the mutant viruses’ genome (Fig. S1).

PCR amplification was employed to confirm that the *meq* and *pp38* genes had been deleted from the MDV genome with the MDV *U_L_33* gene as an internal control since it was not affected by the deletion. As expected, MDV *U_L_33* could be amplified from the Md5BAC (Fig. 1C, lane 11), Md5BAC Δ*pp38* (Fig. 1C, lane 12), Md5BAC Δ*meq* (Fig. 1C, lane 13), Md5BAC Δ*meq*Δ*pp38* (Fig. 1C, lane 14), and Md5BAC Δ*meq*Δ*pp38*-Re (Fig. 1C, lane 15) genomes. However, *meq* was amplified from Md5BAC (Fig. 1C, lane 1), Md5BAC Δ*pp38* (Fig. 1C, lane 2), and Md5BAC Δ*meq*Δ*pp38*-Re (Fig. 1C, lane 5), but not from Md5BAC Δ*meq* (Fig. 1C, lane 3) and Md5BAC Δ*meq*Δ*pp38* (Fig. 1C, lane 4). Similarly, *pp38* could be amplified from Md5BAC (Fig. 1C, lane 6), Md5BAC Δ*meq* (Fig. 1C, lane 8), and Md5BAC Δ*meq*Δ*pp38*-Re (Fig. 1C, lane 10), but not from Md5BAC Δ*pp38* (Fig. 1C, lane 7) and Md5BAC Δ*meq*Δ*pp38* (Fig. 1C, lane 9). The Md5BAC Δ*meq*Δ*pp38* and Md5BAC Δ*meq*Δ*pp38*-Re mutant viruses were also rescued in CEF cells (Fig. 1D). In summary, these results indicate successful generation of *meq* and *pp38* double gene deletion and revertant mutant viruses.

### Double mutant has unimpaired viral replication rate *in vitro* but significantly reduced viral replication rate *in vivo*.

**FIG 2:**
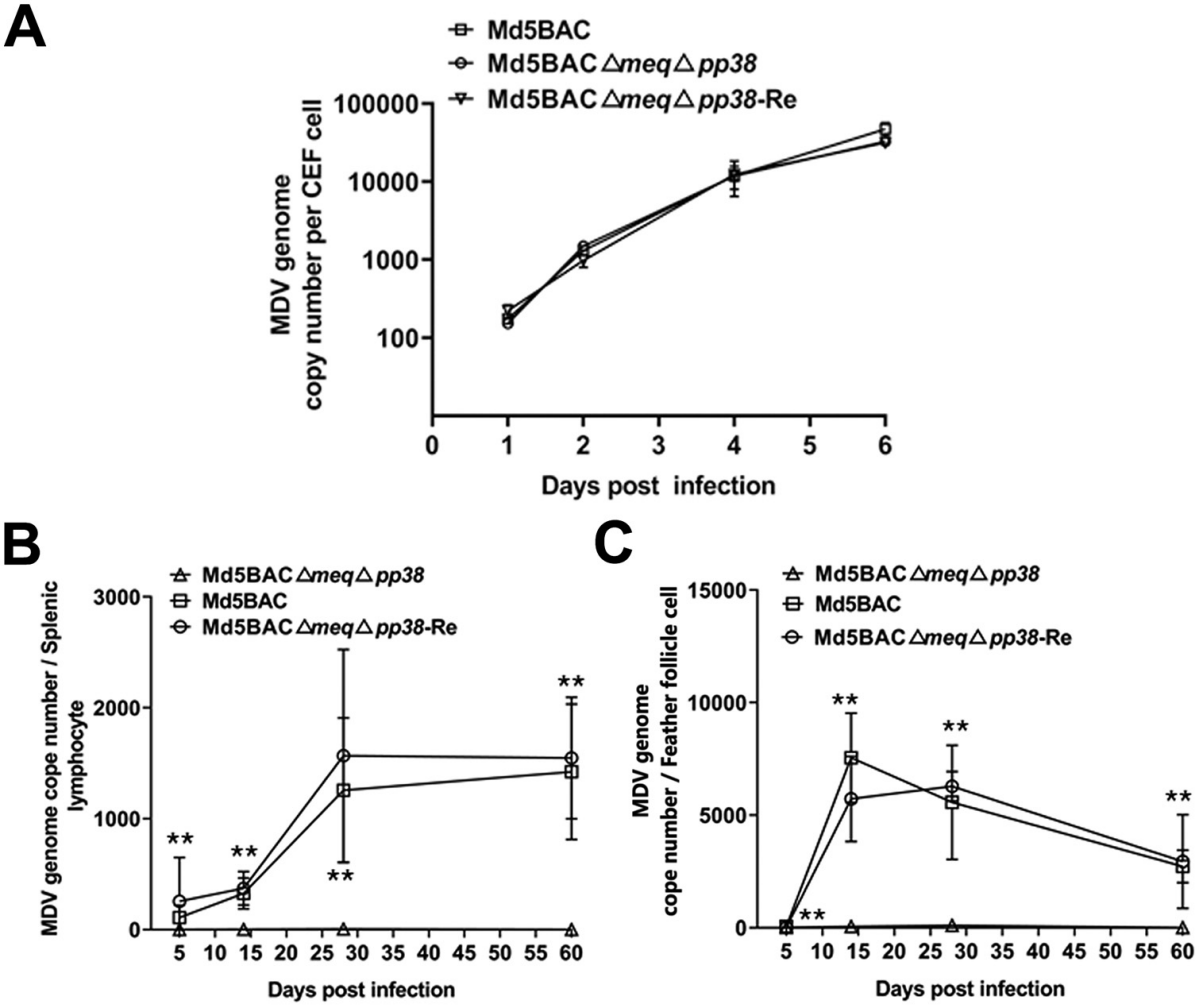
Growth kinetics of the Md5BAC Δ*meq*Δ*pp38* double gene deletion and revertant mutant viruses *in vitro* and *in vivo.* (A) Growth kinetics of recombinant viruses *in vitro* in infected CEF cells on 1, 2, 4, and 6 dpi assessed by qPCR. Each time point represents the mean of triplicates in two independent experiments. For MDV replication measurement *in vivo*, genomic DNA was extracted from splenocytes and FFE cells of 3 chickens in each group, and MDV genome copy number was measured by qPCR. Results are presented as mean MDV genome copy number per splenocyte (B) or FFE (C) cell, with error bars representing standard error of the mean (SEM). The statistical difference between groups were examined by Student's *t* test. **, *P* < 0.01.

Growth kinetics of Md5BAC, Md5BAC Δ*meq*Δ*pp38*, and Md5BAC Δ*meq*Δ*pp38*-Re were compared *in vitro*. As shown in Fig. 2A, there were no significant differences in viral titers at different time points for any virus. These results suggest that the double gene deletion did not affect MDV replication in cell culture.

One-day-old chickens were inoculated with 2,000 PFU of Md5BAC, Md5BAC Δ*meq*Δ*pp38*, or Md5BAC Δ*meq*Δ*pp38*-Re, with control chickens receiving no inoculation. MDV genome copy number was measured at 5, 14, 28, and 60 days post-infection (dpi) using DNA extracted from splenocytes of 3 inoculated chickens. No virus was detected in the negative control group. However, the MDV genome copy number of Md5BAC Δ*meq*Δ*pp38* was significantly lower than the parental Md5BAC or Md5BAC Δ*meq*Δ*pp38*-Re viruses at all time points postinfection (Fig. 2B). MDV usually transmits via bird–bird contact through the feather follicle epithelium (FFE) after infectious viral particles are produced. Investigations of viral genome copy number in the FFE showed significantly lower values for Md5BAC Δ*meq*Δ*pp38* than for the parental Md5BAC or Md5BAC Δ*meq*Δ*pp38*-Re on 5, 14, 28, and 60 dpi (Fig. 2C). These results indicate that simultaneous deletion of *meq* and *pp38* significantly reduced but did not abrogate virus replication in the FFE. Collectively, the results indicate that double deletion of *meq* and *pp38* had no effect on viral replication rate *in vitro* but significantly reduced the rate *in vivo*.

### The double mutant significantly attenuated MDV pathogenesis.

**FIG 3:**
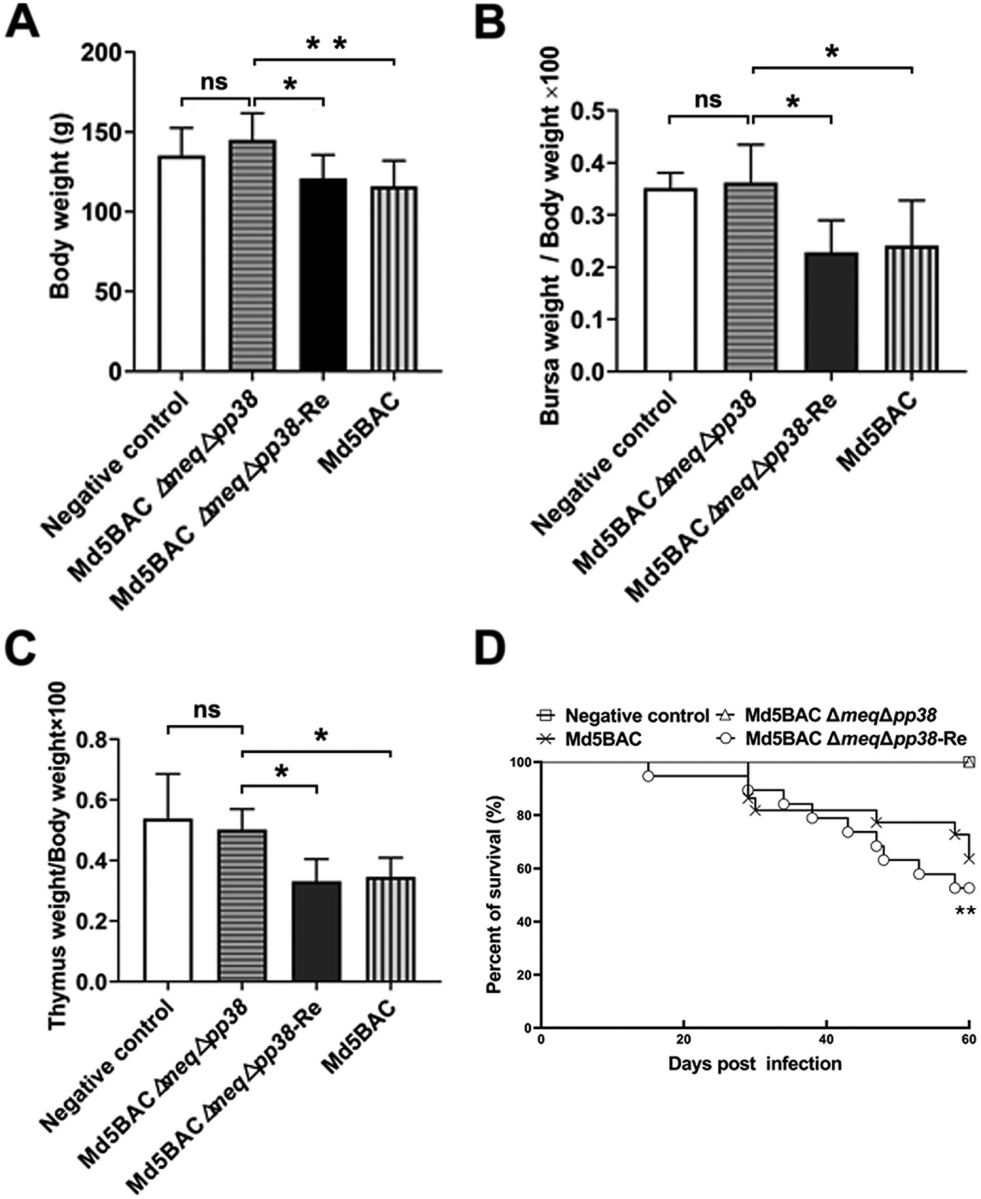
The pathogenic effects of the Md5BAC Δ*meq*Δ*pp38* double gene deletion virus. At day 14 post-challenge, the average body weight was examined (A), and lymphoid organ atrophy was evaluated by mean percentage ratio of bursa (B) or thymus (C) to body weight, with error bars representing standard error of the mean (SEM). Statistical differences were evaluated by Student's *t* test. *, *P < *0.05; **, *P* < 0.01; ns, not significant. (D) Daily mortality of inoculated or negative-control chickens was recorded for 60 days, and percent survival was plotted. Trends of daily survival patterns were assessed by log rank and Wilcoxon test. **, *P < *0.01.

Earlier studies have shown that *meq* deletion abolished oncogenicity but the capacity to induce lymphoid organ atrophy remained (19). *pp38* deletion reduced MDV transformation due to reduced viral replication in lymphoid organs (35, 36). Therefore, we speculated that double *meq* and *pp38* deletion would result in loss of pathogenicity. To test this hypothesis, 1-day-old SPF chickens were inoculated with Md5BAC, Md5BAC Δ*meq*Δ*pp38*, and Md5BAC Δ*meq*Δ*pp38*-Re with uninoculated chickens as a negative control. As expected, the average body weight was significantly reduced in the Md5BAC and Md5BAC Δ*meq*Δ*pp38*-Re groups compared with the Md5BAC Δ*meq*Δ*pp38* group, whereas there was no significant difference between Md5BAC Δ*meq*Δ*pp38* and control groups (Fig. 3A). Additionally, lymphoid organs were examined at 14 dpi. The results showed that Md5BAC and Md5BAC Δ*meq*Δ*pp38*-Re induced severe bursa (Fig. 3B) and thymus atrophy (Fig. 3C) compared with controls. However, relative lymphoid organ weight was not significantly changed in Md5BAC Δ*meq*Δ*pp38* chickens (Fig. 3B and C). Thus, Md5BAC Δ*meq*Δ*pp38* did not cause lymphoid organ atrophy.

All inoculated and control chickens were monitored for mortality for 60 days. MD-associated neurological symptoms began at 26 dpi in the parental Md5BAC group, and 6 out of 20 (30%) died during the experiment. Liver tumors were observed at 36 dpi in the Md5BAC Δ*meq*Δ*pp38*-Re group, and 10 out of 20 (50%) died during the experiment (Fig. 3D and Table 1). Sixty-five percent of Md5BAC-inoculated and Md5BAC Δ*meq*Δ*pp38*-Re-inoculated chickens had MD-specific gross lesions, respectively. However, neither MD-specific mortality nor lesions were apparent in any of the Md5BAC Δ*meq*Δ*pp38*-infected chickens (Table 1). In summary, the viral activity of Md5BAC Δ*meq*Δ*pp38* was fully attenuated.

**TABLE 1:** Comparison of MDV-specific mortality and lesion incidence in SPF chickens

Virus^*a*^	No. of chickens that died/no. tested (%)	No. of chickens with MDV-specific lesions/no. tested (%)
None	0/12 (0)	0/12 (0)
Md5BAC	6/20 (30)	13/20 (65)
Md5BAC Δ*meqΔpp38*	0/16 (0)	0/16 (0)
Md5BAC Δ*meqΔpp38*-Re	10/20 (50)	13/20 (65)

a Chickens were inoculated with 2,000 PFU of the indicated viruses. “None” means no virus inoculation.

### Md5BAC Δ*meqΔpp38* provided better protection than CVI988/Rispens against vv MDV challenge.

**FIG 4:**
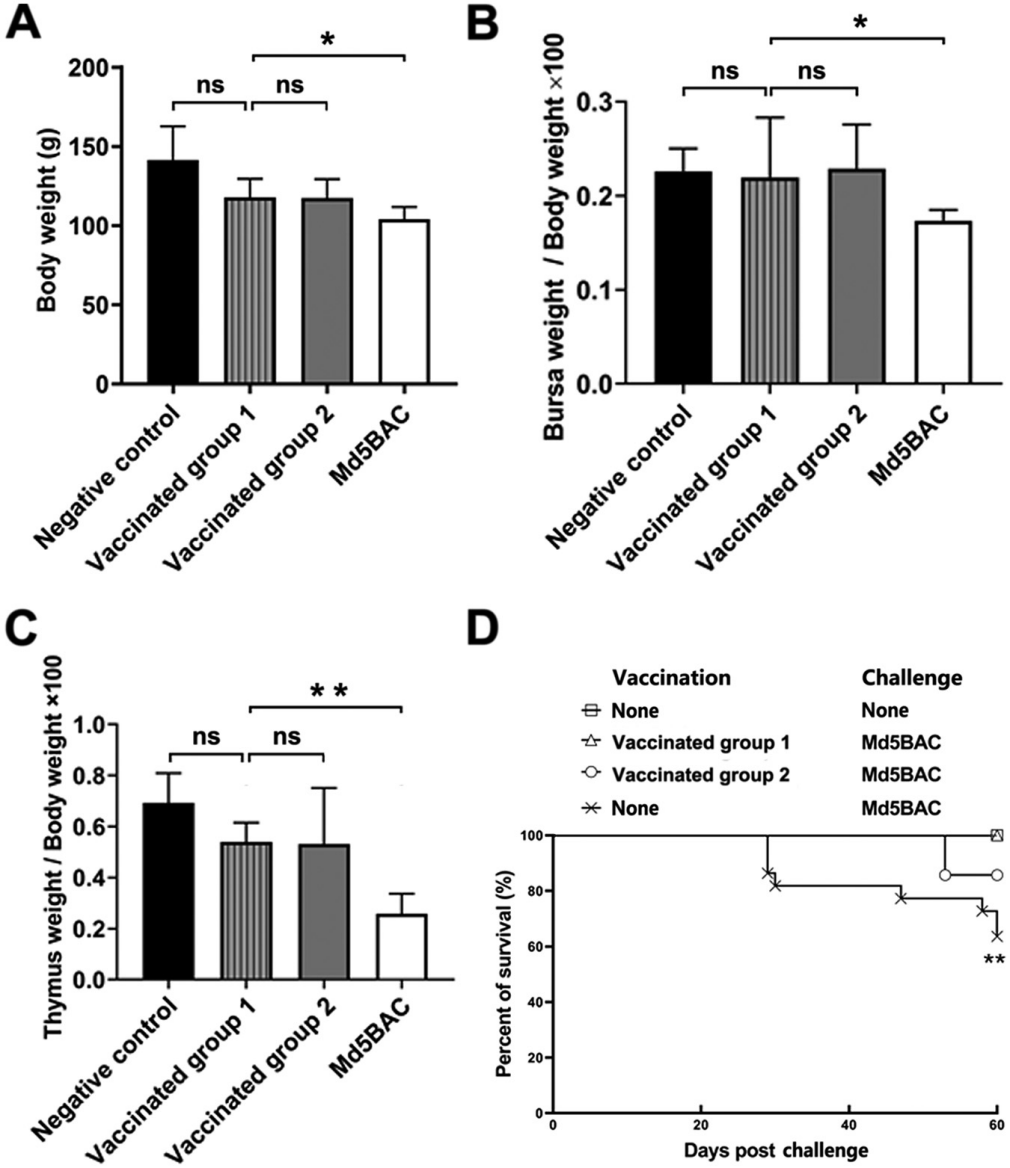
The protective effect of the Md5BAC Δ*meq*Δ*pp38* double gene deletion mutant virus as a vaccine. One-day-old SPF chickens were vaccinated and then challenged with vv MDV at 5 days post-vaccination. At day 14 post-challenge, the average body weight was examined (A), and lymphoid organ atrophy was evaluated by mean percentage ratio of bursa (B) or thymus (C) to body weight, with error bars representing standard error of the mean (SEM). Statistical differences were evaluated by Student's *t* test. *, *P < *0.05; **, *P < *0.01; ns, not significant. (D) Daily mortality of inoculated or negative-control chickens was recorded for 60 days, and percent survival was plotted. Trends of daily survival patterns were assessed by log rank and Wilcoxon test. Vaccinated group 1 represents vaccinated with the Md5BAC Δ*meq*Δ*pp38* mutant strain; Vaccinated group 2 represents vaccinated with the CVI988/Rispens strain.

It is well documented that *meq* deletion viruses give superior protection against highly virulent MDV challenge (16 to 18). We hypothesized that Md5BAC Δ*meq*Δ*pp38* would retain this protection efficacy. One-day-old SPF chickens were vaccinated with Md5BAC Δ*meq*Δ*pp38* (Vaccinated group 1) or CVI988/Rispens (Vaccinated group 2) before challenging with vv MDV at 5 days post-vaccination. As expected, the average body weight was significantly reduced in the Md5BAC group, but not in Md5BAC Δ*meq*Δ*pp38* and CVI988/Rispens vaccine groups (Fig. 4A). In addition, Md5BAC induced significant lymphoid organ atrophy in unvaccinated chickens but not in those vaccinated with Md5BAC Δ*meq*Δ*pp38* or CVI988/Rispens (Fig. 4B and C). All chickens were monitored for gross MD lesions throughout the experiment. Negative-control chickens had no MD-specific gross lesions. In the CVI988/Rispens vaccinated group, MD-specific mortality was 7.7%, but no mortality was observed in the Md5BAC Δ*meq*Δ*pp38* vaccinated group (Fig. 4D). Furthermore, incidence of MD-specific gross tumors and lesions are 7.7% in the CVI988/Rispens vaccinated group, while no tumors or lesions were observed in the Md5BAC Δ*meq*Δ*pp38* vaccinated group (Table 2). Protective index (PI) values were 88.2% for CVI988/Rispens and 100% for Md5BAC Δ*meq*Δ*pp38* vaccinated groups (Table 2). Thus, Md5BAC Δ*meq*Δ*pp38* conferred better protection than CVI988/Rispens against vv MDV challenge.

**TABLE 2:** Protective efficacy of Md5BAC Δ*meqΔpp38* in SPF chickens

Vaccination^*a*^	Challenge^*b*^	Tumors (%)^*c*^	Lesions (%)^*d*^	PI^*e*^
CVI988/Rispens	Md5BAC	1/13 (7.7)	1/13 (7.7)	88.2
Md5BAC Δ*meqΔpp38*	Md5BAC	0/13 (0)	0/13 (0)	100
None	Md5BAC	4/20 (20)	13/20 (65)	N/A^*f*^
None	None	0/12 (0)	0/12 (0)	N/A^*f*^

a Chickens were vaccinated with 2,000 PFU of the indicated viruses.

b Chickens were challenged with 500 PFU Md5BAC at day 5 post-vaccination. “None” means no virus inoculation.

c Tumors (%) = incidence of Marek’s disease specific gross tumors.

d Incidence of Marek’s disease-specific gross lesions.

e PI = protective index.

f N/A = not applicable.

### Transcriptome profiling changes following the Md5BAC Δ*meqΔpp38* and CVI988/Rispens vaccines.

**FIG 5:**
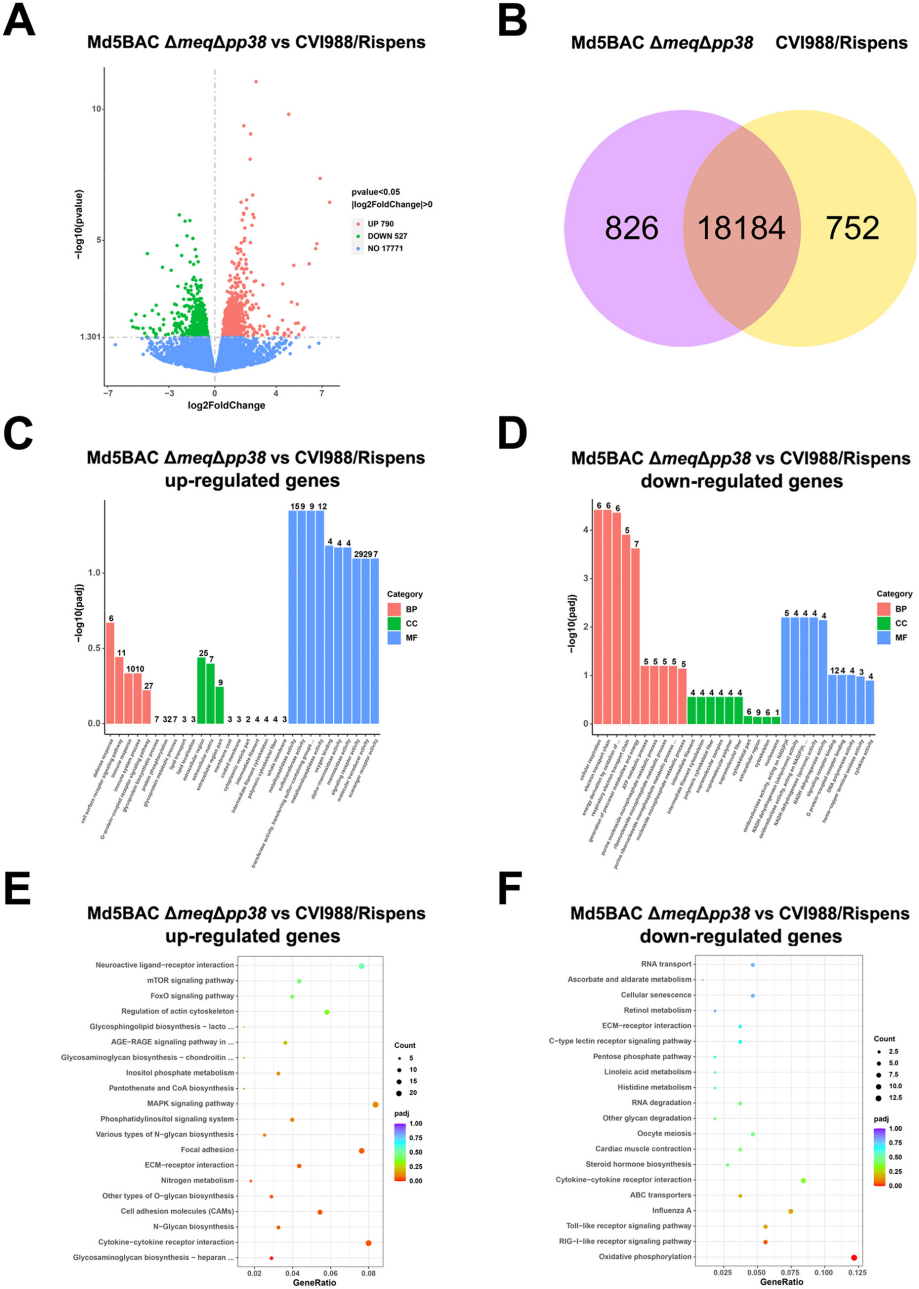
Transcriptome profiling analysis of the MDV-infected chicken spleens at 5 dpi. (A) Volcano diagram identifying the number of significantly regulated genes in MDV-infected chicken spleens comparing Md5BAC Δ*meq*Δ*pp38* and CVI988/Rispens sorted by FDR. (B) Venn diagram showing the number of significantly regulated genes in MDV-infected chicken spleens comparing Md5BAC Δ*meq*Δ*pp38* and CVI988/Rispens. GO analysis showing the cellular responses of upregulated (C) and downregulated genes (D) comparing Md5BAC Δ*meq*Δ*pp38* and CVI988/Rispens. KEGG analysis showing the top 20 signal pathways of m^6^A modified upregulated (E) and downregulated (F) genes between Md5BAC Δ*meq*Δ*pp38* and CVI988/Rispens sorted by *q*-value.

Transcriptome profiling has been used to evaluate the host immune response to MDV infection during the early cytolytic phase by analysis of splenic lymphocytes (37) and was employed in the current study to elucidate why Md5BAC Δ*meq*Δ*pp38* gave superior protection to CVI988/Rispens. Transcriptome profiling was performed during Md5BAC Δ*meq*Δ*pp38* and CVI988/Rispens lytic replication at 5 dpi, using uninfected spleen samples as negative control. Md5BAC Δ*meq*Δ*pp38* infection induced upregulation of 382 genes and downregulation of 391 genes compared with controls (*P < *0.05, Log_2_^Fold change^ > 0; Fig. S2A). CVI988/Rispens infection induced upregulation of 1,582 genes and downregulation of 2,203 genes (Fig. S2B). Seven hundred ninety genes were upregulated and 527 genes downregulated in Md5BAC Δ*meq*Δ*pp38*-infected compared with CVI988/Rispens-infected spleens (*P < *0.05, Log_2_^Fold change^ > 0; Fig. 5A). Further analysis revealed 18,184 genes that were regulated by both Md5BAC Δ*meq*Δ*pp38* and CVI988/Rispens, while 826 were specific to Md5BAC Δ*meq*Δ*pp38* and 752 genes were specific to CVI988/Rispens (Fig. 5B).

The DAVID bioinformatics database was used to identify GO terms associated with the cellular responses to Md5BAC Δ*meq*Δ*pp38* and CVI988/Rispens, and different biological processes (BP), cellular components (CC), and molecular functions (MF) were found to be induced (Fig. S3). Md5BAC Δ*meq*Δ*pp38* induced upregulated defense response, immune response, and immune system processes (BP), extracellular region, extracellular matrix and extracellular region part (CC), metallopeptidase activity, signaling receptor activity, and molecular transducer activity (MF) compared with CVI988/Rispens (Fig. 5C). Meanwhile, the Md5BAC Δ*meq*Δ*pp38* induced significantly downregulated cellular respiration and electron transport chain (BP), intermediate filament, intermediate filament cytoskeleton and polymeric cytoskeletal fiber (CC), and NADH dehydrogenase activity, signaling receptor binding and DNA polymerase activity (MF) compared with CVI988/Rispens (Fig. 5D). It may be concluded that Md5BAC Δ*meq*Δ*pp38* infection induced different cellular responses compared with CVI988/Rispens.

Differences between Md5BAC Δ*meq*Δ*pp38* and CVI988/Rispens were also apparent from pathway analysis (Fig. S4). Md5BAC Δ*meq*Δ*pp38* upregulated genes associated with immune responses, including cytokine-cytokine receptor interaction, cell adhesion molecules (CAMs), focal adhesion, and ECM-receptor adhesion (Fig. 5E), and downregulated those correlated with RIG-I-like receptor signaling pathway and oxidative phosphorylation compared with CVI988/Rispens (Fig. 5F). Collectively, these results indicate that Md5BAC Δ*meq*Δ*pp38* and CVI988/Rispens may have distinct immunological mechanisms when used as vaccines.

## DISCUSSION

MDV reduces the performance of innate and adaptive immunity in chickens (38, 39), and vaccination prevents and controls disease occurrence (3, 4). CVI988/Rispens is the most effective vaccine currently available, but as more highly virulent viruses emerge, vaccine-induced immunity may be breached (7, 40, 41). Our previous studies have shown that *vIL8* deletion in the 686BAC Δ*meq* virus eliminated lymphoid organ atrophy and protected against vv+ MDV challenge (9). The search for more efficacious and safe vaccine candidates has led us to explore the impact of other genes. Deletion of *pp38* has been shown to reduce pathogenesis, especially lymphoproliferative lesions (36). The hypothesis that deletion of *pp38* in MDV Δ*meq* may reduce lymphoid organ atrophy but still retain protective efficacy was tested by the generation of a double gene deletion mutant, Md5BAC Δ*meq*Δ*pp38*. Replication of Md5BAC Δ*meq*Δ*pp38* was similar to that of the parental virus *in vitro* (Fig. 2A) but was impaired *in vivo* (Fig. 2B), suggesting altered properties resulting from *pp38* deletion. Furthermore, replication of Md5BAC Δ*meq*Δ*pp38* did not induce lymphoid organ atrophy or mortality, suggesting that the *pp38* deletion that contributed to the full attenuation of the *meq* deletion mutant virus should prove safe as a potential MDV vaccine (Fig. 3B and C). MDV normally undergoes reactivation in the FFE, leading to the shedding of infectious virus into the environment to complete the life cycle (42). Neither deletion of *pp38* (34) nor of *meq* (15) interfered with viral replication in the FFE, suggesting that these genes are not essential for MDV transmission. This suggestion was confirmed by Md5BAC Δ*meq*Δ*pp38* infection (Fig. 2C).

Immune protective effects of Md5BAC Δ*meq*Δ*pp38* and CVI988/Rispens were compared by the assessment of lymphoid organ atrophy after challenge with vv MDV *in vivo*. Neither vaccine-treated group showed any lymphoid organ atrophy (Fig. 4B and C). It should be noted that most immunized chickens resisted Md5BAC attack, suggesting the generation of effective immunity (Table 2). It has been suggested that an effective MDV vaccine would have to replicate to sufficient levels to stimulate an immune response, enter the latency phase, and be able to reactivate from time to time to sustain and boost the immune response (43, 44). Md5BAC Δ*meq* has previously been shown to establish long-term protection since it replicates efficiently in the initial cytolytic phase (16). The current study found Md5BAC Δ*meq*Δpp38 to replicate slowly but to remain capable of inducing a vigorous host immune response and providing excellent protection (Fig. 4 and Table 2). These are all promising qualities for an effective vaccine.

The protective mechanism of an MDV vaccine remains to be elucidated, but previous studies have demonstrated that both pathogenic and nonpathogenic strains stimulate differential gene expression *in vitro* and *in vivo* (37, 45). CVI988/Rispens inoculation caused altered transcriptome profiles in splenic lymphocytes compared with pathogenic virus in the early cytolytic infection phase (37). Interestingly, Md5BAC Δ*meq*Δ*pp38* was found to confer superior protection to CVI988/Rispens (Fig. 4 and Table 2). High-throughput RNA sequencing of splenic transcriptomes during the early cytolytic infectious phase indicated up- and downregulation of genes in different cellular responses and pathways by Md5BAC Δ*meq*Δ*pp38* (Fig. 5A and B). Md5BAC Δ*meq*Δ*pp38* upregulated defense response, immune response, immune system processes, and immunity-associated cytokine–cytokine receptor interaction (Fig. 5E). Alternatively, CVI988/Rispens is a cell culture passage attenuated virus and the gold standard among MDV vaccines. Attempts have been made to attenuate highly virulent MDV strains by serial passage in cell culture (46). Resulting single nucleotide polymorphisms (SNPs) and gene deletions (47) may correlate with vaccine efficiency. The current *meq* and *pp38* double gene deletion virus contained no unexpected rearrangements in the viral genome (Fig. 1, and Fig. S1 in the supplemental material); however, as a cytoplasmic protein phosphorylated by U_S_3, pp38 is involved in the early cytolytic replication of MDV (27, 33). A recent study indicated that Us3 promotes MDV replication through blocking interferon β (IFN-β) by targeting IFN regulatory factor 7 (IRF7) (48). In addition, Us3 also regulates the phosphorylation of Meq (30), which can antagonize the innate cGAS-STING signaling pathway to mediate immune evasion (49). Overall, we speculate that both pp38 and Meq are involved in the immune evasion process once phosphorylated. Thus, the double deletion mutant confers good immunological protection against highly virulent MDV infection. Collectively, these data suggest distinct immunological mechanism between Md5BAC Δ*meq*Δ*pp38* and CVI988/Rispens when used as vaccines.

Previous studies have shown distinctive host transcriptomic profiling changes induced by different MDV strains, and viral and cellular microRNAs (miRNA) are known to regulate gene expression (50). Recently, the RNA N6-methyladenosine (m^6^A) modification emerged as an additional layer of gene regulation at post-transcriptional level and regulates mRNA metabolism, which may account for viral and cellular transcriptomic control (51, 52). Importantly, the m^6^A modification may regulate the immune system to facilitate viral replication (53). We have also reported alterations in transcriptome-wide m^6^A modification in chicken lncRNAs and circRNAs (54, 55). Recently, we found spatiotemporal changes of mRNA m^6^A modification in distinct MDV infection phases (unpublished data). Thus, it is possible that Md5BAC Δ*meq*Δ*pp38* may affect viral and cellular gene expression via the miRNA and m^6^A modification regulation.

In conclusion, the double gene deletion, Md5BAC Δ*meq*Δ*pp38* strain, had unimpaired *in vitro* replication but significantly reduced replication *in vivo*. In addition, Md5BAC Δ*meq*Δ*pp38* abolished the lymphoid organ atrophy induced by Md5BAC Δ*meq*. The double deletion virus gave better protection against vv MDV challenge compared with CVI988/Rispens. Thus, our study indicates a valuable candidate for new MDV vaccine development.

## MATERIALS AND METHODS

### Experimental chickens, cells, and viruses.

Specific pathogen free (SPF) chickens and eggs were purchased from Boehringer Ingelheim, Beijing, China. Chicken embryonic fibroblasts (CEFs) were isolated from 9-day-old chicken embryos, as previously described (21), prepared for *in vitro* experiments, and cultured in Dulbecco's modified Eagle’s medium with 5% fetal bovine serum (FBS) at 37°C. The *meq* and *pp38* deletion and revertant mutant viruses were generated from an Md5BAC strain (21).

### Construction of *meq* and *pp38* double deletion mutant virus.

A two-step Red-mediated recombination procedure was performed to delete *meq* and *pp38* genes from Md5BAC, individually or in combination, as previously described (56). In brief, the *Kana^R^-I-SceI* cassette was amplified from pEPkana-S and electroporated into E. coli containing Md5BAC. The *Kana* sequence was deleted by addition of arabinose to generate a *meq* deletion mutant construct, Md5BAC Δ*meq*, as previously described (19). Md5BAC Δ*meq* was used as the backbone to generate the *meq* and *pp38* double deletion mutant, Md5BAC Δ*meq*Δ*pp38*, by the same procedure described above. The revertant mutant construct was generated by reinserting the deleted *meq* or *pp38* sequences into Md5BAC Δ*meq*Δ*pp38* to produce Md5BAC Δ*meq*Δ*pp38-*Re. All mutant viruses were screened by PCR, DNA sequencing, and restriction fragment length polymorphism (RFLP) assay to confirm the deletion of *meq* and *pp38* and exclude the possibility of unexpected mutations. Primers used to construct all mutant BAC clones are shown in Table S1. Gene deletion and revertant constructs were transfected into CEFs to produce recombinant viruses.

### Immunofluorescence (IFA) assay.

IFA was carried out as previously described with simple modifications (31). Briefly, CEFs infected with MDV were washed carefully with phosphate-buffered saline (PBS) and fixed with an ice-cold mixture of acetone and methanol (3:2) for 10 min. After blocking for 2 h with 5% skimmed milk, CEFs were incubated with MDV gB monoclonal antibody (1:500) for 1 h, given 3 × 15 min washes, and incubated with goat anti-mouse fluorescein isothiocyanate (FITC)-labeled secondary antibody (KPL, Gaithersburg, MD, USA) for 1 h. CEFs were given 3 × 15 min washes and imaged under a fluorescence microscope.

### *In vitro* growth kinetics.

*In vitro* growth kinetics were determined for MDVs, as previously described (21, 57 to 59). Briefly, CEFs were seeded onto 60-mm plates and inoculated with 100 PFU of each virus. On days 1, 3, and 5 post-inoculation, CEFs were collected and MDV genome copy number was measured for virus titration. The cycle threshold (Ct) values for the chicken ovotransferrin (*OVO*) gene and the viral *ICP4* gene were determined by qPCR and a standard curve equation calculated according to the linear relationship between the logarithm of genome copy number and Ct. Viral genome copy number was calculated to compare the *in vitro* growth kinetics and replication of the recombinant and parental viruses, as previously described (21, 57 to 59).

### Pathogenesis of Md5BAC Δ*meqΔpp38* mutant viruses in SPF chickens.

One-day-old SPF chickens were wing-banded and randomly sorted into 4 experimental groups for inoculation with 2,000 PFU of parental Md5BAC; 2,000 PFU Md5BAC Δ*meq*Δ*pp38*; 2,000 PFU Md5BAC Δ*meq*Δ*pp38*-Re; or no inoculation (negative control).

Three chickens were randomly selected from each experimental group, weighed, and euthanized at 14 dpi. Thymus and bursa were weighed to evaluate lymphoid organ atrophy. Results are presented as mean percentage ratios of lymphoid organ weight to body weight.

Daily mortality was recorded for each experimental group for 60 days to compare the pathogenic properties of parental Md5BAC, Md5BAC Δ*meq*Δ*pp38*, and Md5BAC Δ*meq*Δ*pp38*-Re. All chickens that died or were euthanized were necropsied and examined for MD-associated gross tumors and lesions.

### Vaccine protection experiment.

One-day-old SPF chickens were either unvaccinated or subcutaneously vaccinated with 2,000 PFU of Md5BAC Δ*meq*Δ*pp38* (Vaccinated group 1) or CVI988/Rispens (Vaccinated group 2). Five days later, all chickens were challenged subcutaneously with 500 PFU of Md5BAC. Chickens that died or survived to 60 days post-challenge were necropsied and examined for MD-associated gross tumors and lesions. Vaccine protection efficacy was expressed as protective index (PI), as previously described (9, 16).

### MDV genome copy number measurement.

Three chickens from each group were euthanatized at 5, 14, 28, or 60 dpi, and spleen samples were collected. Genomic DNA was extracted from splenocytes using phenol-chloroform and MDV genome copy number measured by quantitative PCR (qPCR) using primers specific for *ICP4* and *OVO* genes, as previously described (21, 57). All qPCR assays were carried out in a Bio-Rad iCycler iQ Multicolor Real-Time Detection System, using iTag SYBR supermix buffer (Bio-Rad, USA). Results are presented as the ratio of *ICP4* copy number to *OVO* copy number, with error bars representing standard error of the mean (SEM).

### RNA extraction and transcriptome sequencing.

Total RNA was isolated from chicken spleens, and quality and integrity were assessed by RNA Nano 6000 assay kit using the Bioanalyzer 2100 system (Agilent Technologies, CA, USA).

mRNA was purified from total RNA using poly T oligo-attached magnetic beads and fragmented with divalent cations and elevated temperature in First Strand Synthesis Reaction Buffer (5×). First-strand cDNA was synthesized using random hexamer primers and M-MuLV reverse transcriptase (RNase H). Second-strand cDNA synthesis was performed using DNA polymerase I and RNase H. Remaining overhangs were converted into blunt ends via exonuclease/polymerase activities. 3′ ends of DNA fragments were adenylated and adaptor with hairpin loop structures ligated to prepare for hybridization. Library fragments were purified with AMPure XP system (Beckman Coulter, Beverly, USA), and cDNA fragments of 370 to 420 bp were selected. PCR was performed with Phusion High-Fidelity DNA polymerase. PCR products were purified (AMPure XP system), and library quality was assessed on the Agilent Bioanalyzer 2100 system. Raw data (raw reads) in fastq format were processed through in-house perl scripts to remove reads containing adaptor, reads containing poly N, and low-quality reads from raw data. Q20, Q30, and GC contents of the clean data were calculated. All downstream analyses were based on clean high-quality data with clean reads being mapped to reference chicken genome (Gallus_gallus.GRCg6a.cds.all.fa.gz).

### Gene expression analysis.

Analysis of differentially expressed genes in two conditions/groups (two replicates per condition) was performed using the DESeq2R package (1.20.0) using a model based on the negative binomial distribution. The resulting *P* values were adjusted using Benjamini and Hochberg’s approach for controlling the false discovery rate (FDR). Genes with an adjusted *P* value less than 0.05 found by DESeq2 were considered to be differentially expressed.

### GO and KEGG pathway analysis.

Gene ontology (GO) enrichment analysis of differentially expressed genes was conducted by ClusterProfiler R package, in which gene length bias was corrected. GO terms with corrected *P* value less than 0.05 were considered significantly enriched within the grouping of differentially expressed genes. Kyoto Encyclopedia of Genes and Genomes (KEGG) is a database resource for understanding high-level functions and utilities of the biological system, such as the cell, the organism, and the ecosystem, from molecular-level information, especially large-scale molecular data sets generated by genome sequencing and other high-throughput experimental technologies (http://www.genome.jp/kegg/). ClusterProfiler R package was used to assess enrichment of differentially expressed genes in KEGG pathways.

### Statistical analysis.

All the experiments were conducted, and samples collected, in triplicate. Student's *t* test was used for statistical analysis of RT-qPCR results. One-way ANOVA was used for statistical analysis of viral replication-associated data. Animal survival curves were compared using log-rank and Wilcoxon tests. Data were analyzed, and results were presented as means with standard errors. All statistical analyses were plotted with GraphPad Prism 8 (GraphPad Software, LLC. San Diego, CA). A *P* value of less than 0.05 was considered to indicate statistical significance.

### Ethics statement.

The animal study was reviewed and approved by the ethics and animal welfare committee of Henan Agricultural University following the national *Guide for the Care and Use of Laboratory Animals* (approval number: SYXK-YU-2021-0003).

### Data availability.

All data generated or analyzed during this study are included in this submitted manuscript. The data sets generated and/or analyzed in this study are available in the NCBI repository (https://www.ncbi.nlm.nih.gov/geo/). The data are accessible via NCBI GEO submission ID GSE208411.
