# Supplementary material for: Fully Attenuated meq and pp38 Double Gene Deletion Mutant Virus Confers Superior Immunological Protection against Highly Virulent Marek’s Disease Virus Infection
Source: Microbiol Spectr. 2022 Nov 9;10(6):e02871-22. doi: 10.1128/spectrum.02871-22 (PMC9769808; doi:10.1128/spectrum.02871-22)
Supplement: Supplemental file 1 — Fig. S1 to S4; Table S1. Download spectrum.02871-22-s0001.pdf, PDF file, 1.4 MB [file spectrum.02871-22-s0001.pdf]

SUPPLEMENTAL MATERIALS

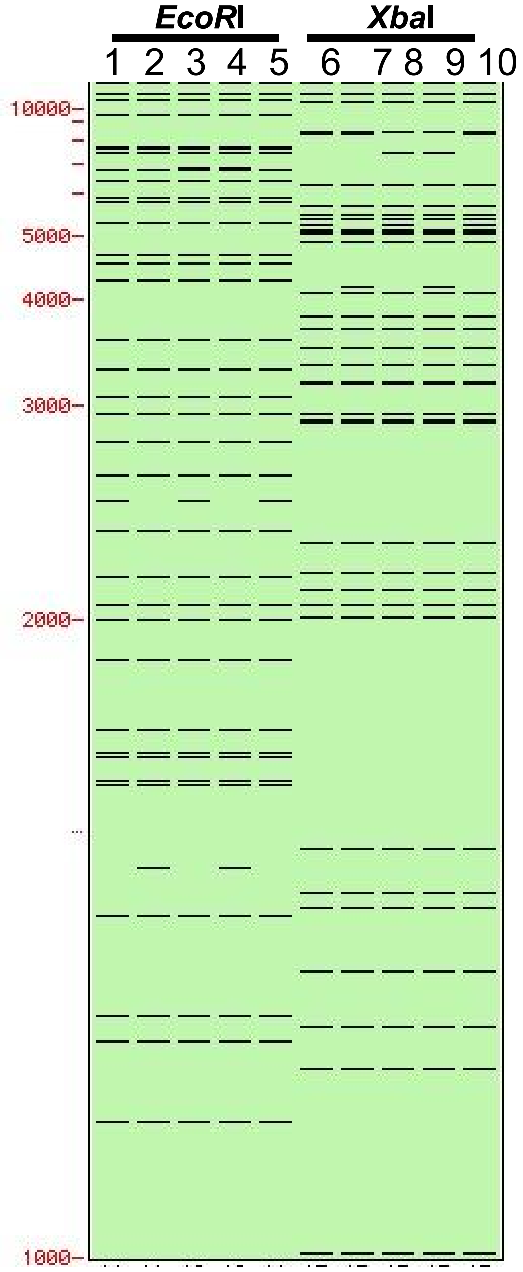

Supplementary Figure 1. Computational prediction of RFLP result by Snapgene software; lanes 1 and 6: Md5BAC; lanes 2 and 7: Md5BAC△*meq*; lanes 3 and 8: Md5BAC△*pp38*; lanes 4 and 9: Md5BAC △*meq*△*pp38*; lanes 5 and 10: Md5BAC △*meq*△*pp38*-Re.

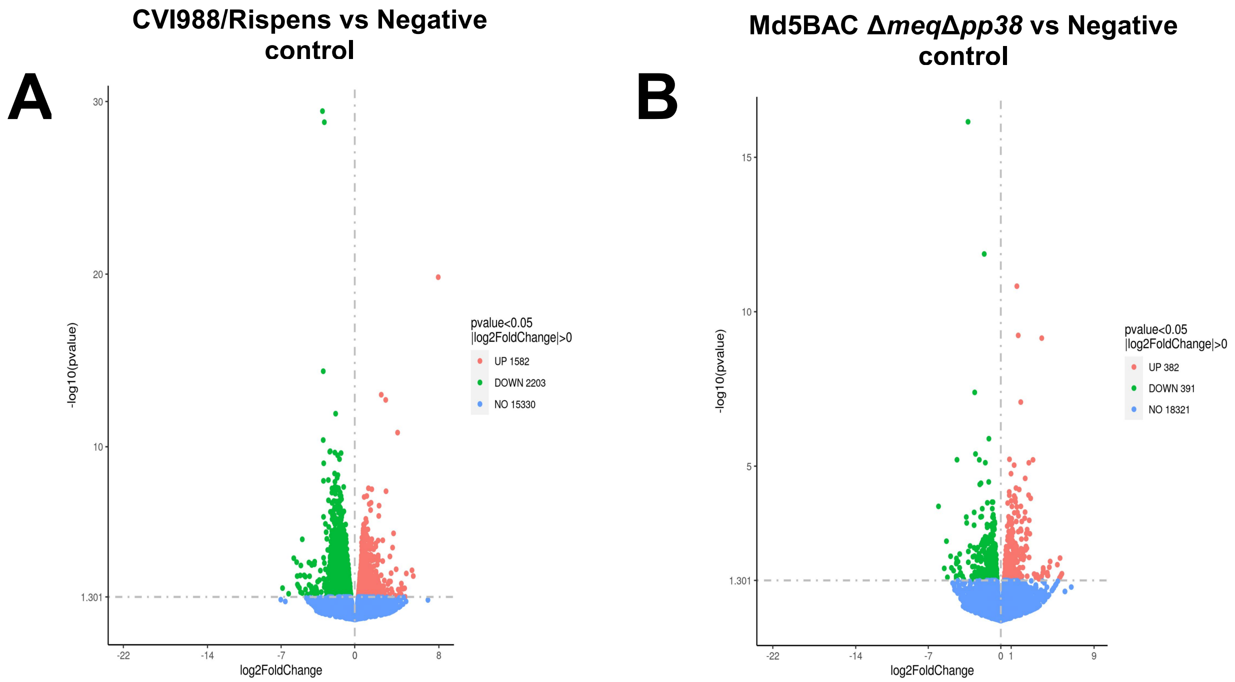

Supplementary Figure 2. Volcano diagram identifying the number of significantly regulated genes in MDV infected chicken spleens comparing Md5BAC △*meq*△*pp38* versus negative control **(A)** and CVI988/Rispens versus negative control **(B)** sorted by FDR.

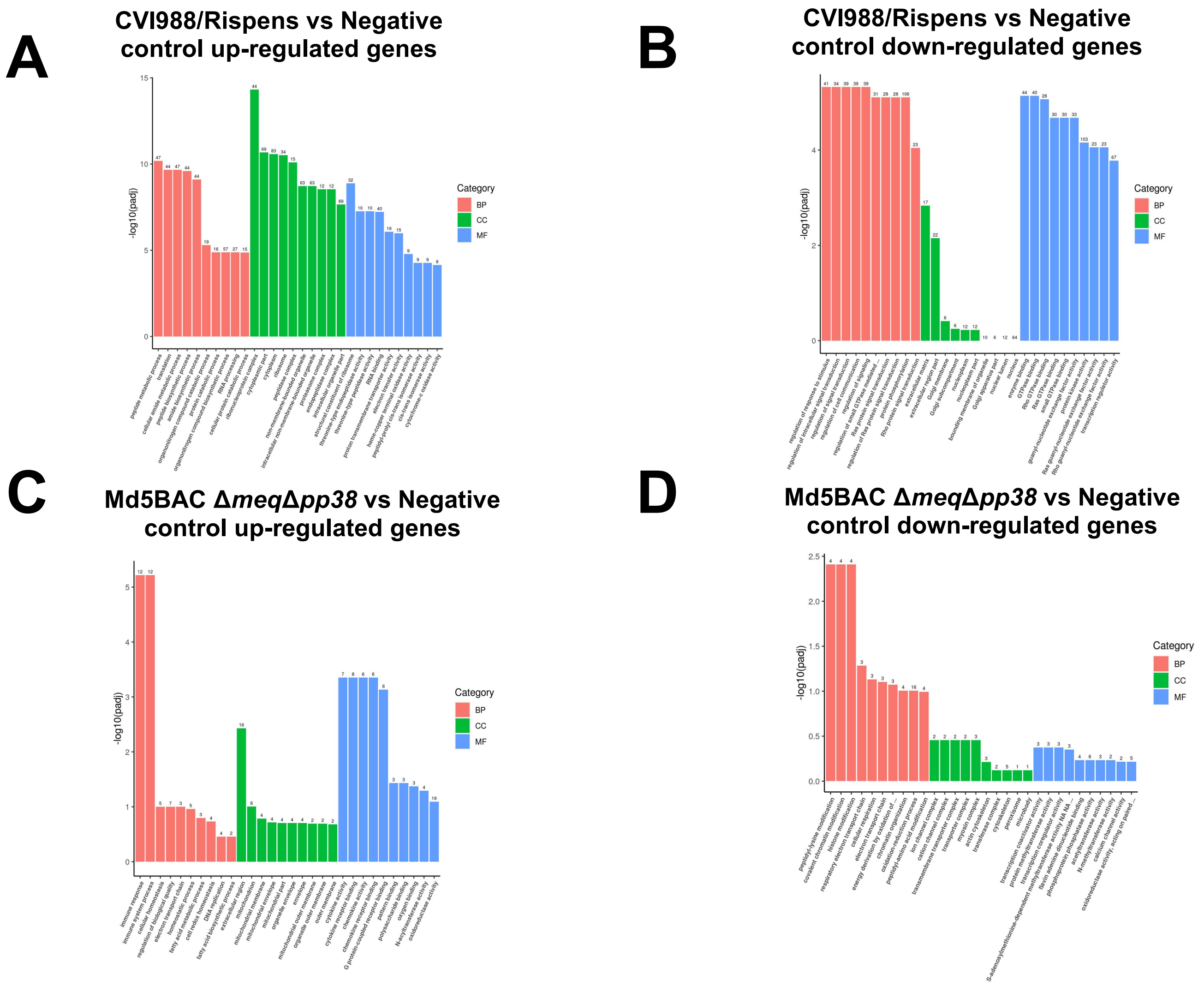

Supplementary Figure 3. GO analysis showing cellular response up-regulated **(A)** and down-regulated genes **(B)** comparing CVI988/Rispens and negative control. GO analysis shows the cellular response up-regulated **(C)** and down-regulated genes **(D)** comparing Md5BAC △*meq*△*pp38* and negative control.

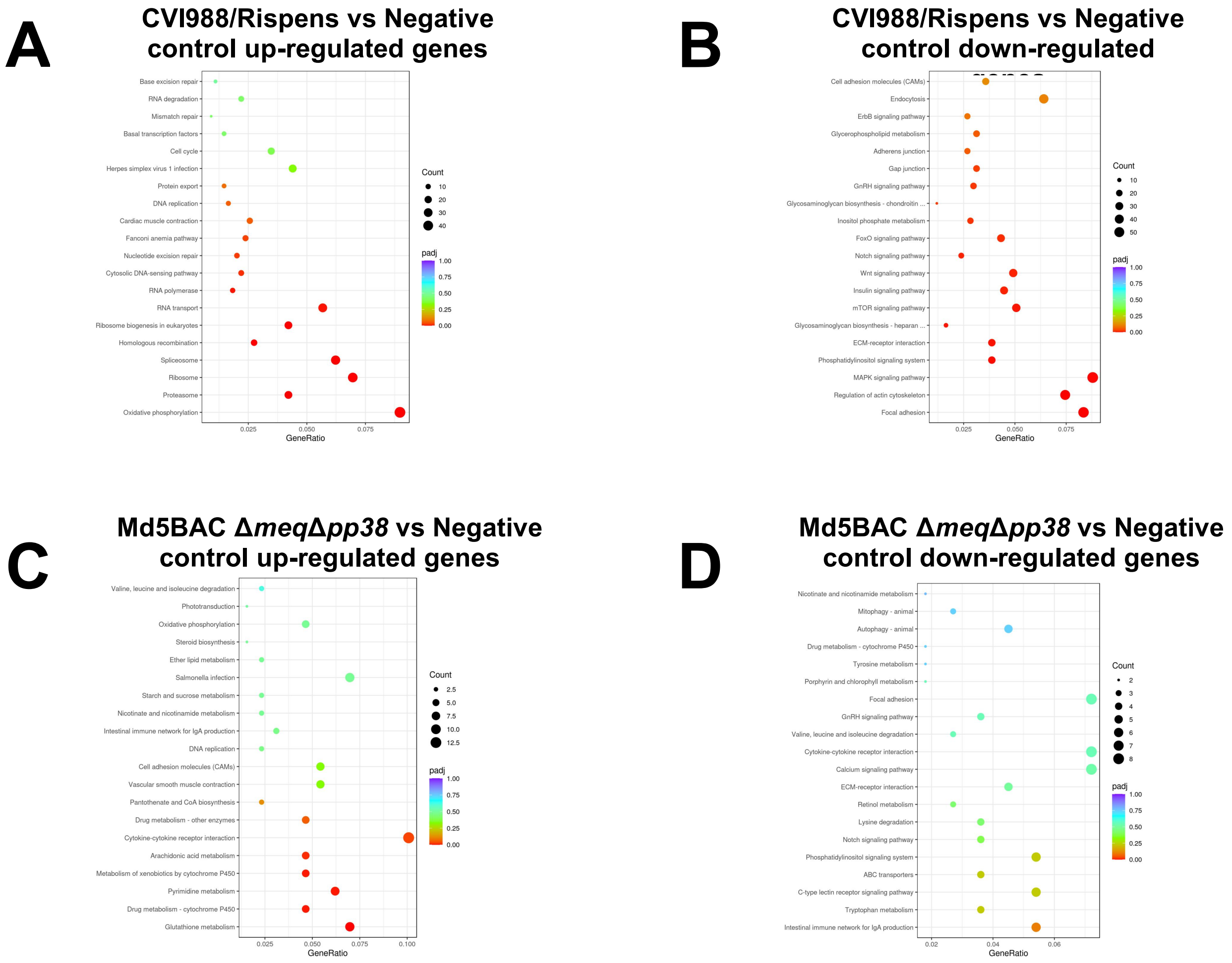

Supplementary Figure 4. KEGG analysis showing the top 20 signal pathways of m^{6}A modified up-regulated **(A)** and down-regulated **(B)** genes comparing CVI988/Rispens and negative control sorted by q-value. KEGG analysis showing the top 20 signal pathways of m^{6}A modified up-regulated **(C)** and down-regulated **(D)** genes comparing Md5BAC △*meq*△*pp38* and negative control sorted by q-value.

Supplementary Table 1. List of primers used in the experiment

Primers	Sequence (5'-3')	Purpose
pp38-Kana^R-F	**TTCGCTTAATCTCCGCCTCCAACATCG**<br>**GGTACGGCTACACTGTATTAAAT**AGGA<br>TGACGACGATAAGTAGGG	Amplification of Kana^R cassette with MDV sequences flanking *pp38* gene
pp38-Kana^R-R	**CCCGCACCGCACGCTTTGCTCGTCCCC**<br>**GCGTGCAAGATCGGCAGGGGGTGATT**<br>**TAATACAGTGTAGCCGTACCCGATGTT**<br>**GGAGGCGGAGATTAAGCGAA**CAACCA<br>ATTAACCAATTCTGATTAG	
meq-Kana^R-F	**CTTGCAGGTGTATACCAGGGAGAAGG**<br>**CGGGCACGGTACAGGTGTAAAGAG**AG<br>GATGACGACGATAAGTAGGG	Amplification of Kana^R cassette with MDV sequences flanking *meq* gene
meq-Kana^R-R	**AGAAACATGGGGCATAGACGATGTGC**<br>**TGCTGAGAGTCGCAATGCGGATCACTC**<br>**TTTACACCTGTACCGTGCCCGCCTTCT**<br>**CCCTGGTATACACCTGCAAG**CAACCAA<br>TTAACCAATTCTGATTAG	
meq-F	CCGCACACTGATTCCTAG	Amplification of *meq* gene
meq-R	CCTTTATGTTGATCTTCCCG	
pp38-F	GATCGGATCCTTCGCTTAATCTCCGCCTC	Amplification of *pp38* gene
pp38-R	GATCGATATCGAACTTCTTCGCCTGATC	
U_L33-F	GATCAAGCTTCATCACTCTGAACATCC	Amplification of U_L33 gene
U_L33-R	GATCGGATCCCAACCAGAAAATGACTC	

"F" means forward primer; "R" means reverse primer; For primers pp38-kana^R-F, pp38-kana^R-R, meq-kana^R-F and meq-kana^R-R, underlined sequences indicate the sequences used to amplify the Kana^R gene cassette. Sequences in bold indicate MDV genome sequences flanking *pp38* or *meq* genes.
